# Event Prediction Model Considering Time and Input Error Using Electronic Medical Records in the Intensive Care Unit: Retrospective Study

**DOI:** 10.2196/26426

**Published:** 2021-11-04

**Authors:** MinDong Sung, Sangchul Hahn, Chang Hoon Han, Jung Mo Lee, Jayoung Lee, Jinkyu Yoo, Jay Heo, Young Sam Kim, Kyung Soo Chung

**Affiliations:** 1 Department of Biomedical Systems Informatics Yonsei University College of Medicine Seoul Republic of Korea; 2 AITRICS. Inc Seoul Republic of Korea; 3 Division of Pulmonology Department of Internal Medicine National Health Insurance Service Ilsan Hospital Goyang-si Republic of Korea; 4 Korea Advanced Institute of Science and Technology Daejeon Republic of Korea; 5 Division of Pulmonology Department of Internal Medicine Yonsei University Health System Seoul Republic of Korea

**Keywords:** machine learning, critical care, prediction model, intensive care unit, mortality, AKI, sepsis

## Abstract

**Background:**

In the era of artificial intelligence, event prediction models are abundant. However, considering the limitation of the electronic medical record–based model, including the temporally skewed prediction and the record itself, these models could be delayed or could yield errors.

**Objective:**

In this study, we aim to develop multiple event prediction models in intensive care units to overcome their temporal skewness and evaluate their robustness against delayed and erroneous input.

**Methods:**

A total of 21,738 patients were included in the development cohort. Three events—death, sepsis, and acute kidney injury—were predicted. To overcome the temporal skewness, we developed three models for each event, which predicted the events in advance of three prespecified timepoints. Additionally, to evaluate the robustness against input error and delays, we added simulated errors and delayed input and calculated changes in the area under the receiver operating characteristic curve (AUROC) values.

**Results:**

Most of the AUROC and area under the precision-recall curve values of each model were higher than those of the conventional scores, as well as other machine learning models previously used. In the error input experiment, except for our proposed model, an increase in the noise added to the model lowered the resulting AUROC value. However, the delayed input did not show the performance decreased in this experiment.

**Conclusions:**

For a prediction model that was applicable in the real world, we considered not only performance but also temporal skewness, delayed input, and input error.

## Introduction

Since intensive care resources are always limited, and better resource allocation leads to better outcomes [1], conventional scores such as Acute Physiology And Chronic Health Evaluation (APACHE) [2], Simplified Acute Physiology Score (SAPS) [3], and Mortality Probability Models (MPMs) [4] have been used to predict the outcome of patients admitted to the intensive care unit (ICU). However, because the status of patients in the ICU changes rapidly, predicting adverse events and clinical complications, which are a major cause of mortality and poor outcomes, can buy some time to intervene and change the natural disease course [5,6]. Although conventional scores are widely used, these scores use only the features of patients at admission, and there have been many attempts to develop prediction models using time-series data.

With the increased use of electronic medical records (EMRs) [7] and artificial intelligence (AI), many AI models have been developed to predict events in the health care domain [8], and the intensive care domain is no exception. Additionally, the ICU generates many different kinds of frequently measured data. Thus, many models have been developed with a focus on ICU data [9-13].

Previous models were developed using retrospective EMR data. To apply these models in the real world, two points should be considered. The model should know more than whether an event will occur within the predicted time frame. In most studies, the distribution of event occurrence within the follow-up time is skewed to one side [5,9]. We defined this phenomenon as “temporal skewness,” which means more true-positive samples occur when the prediction time is getting closer to the actual event onset time. In particular, the performance metrics of a rapid response team are directly linked to a guarantee of temporal dependence regarding treatment intervention feasibility, similar to the 1-hour bundle suggested by sepsis treatment guidelines. Second, medical record data are often entered incorrectly, delayed, or even frequently missed in the field during patient care [14]. These errors should affect any real-time prediction model. Even if humans were replaced by an internet of medical things (IoMT) sensor [15], these sensors can generate noise in the data and transactions can be delayed. Thus, the model should be robust in consideration of these input errors.

Therefore, the prediction model using EMRs should achieve the following: (1) correction of temporal skewness and (2) robustness against delayed input and data input errors. Herein, we developed a novel prediction model using deep learning techniques that can be clinically applied to achieve the two abovementioned points.

## Methods

### Study Participants and the Development Cohort

We retrospectively enrolled adult patients who were admitted to the ICU from 2013 to 2017 at the Severance Hospital, a tertiary academic medical center in South Korea that includes medical, medicosurgical, neurological, cardiac surgery recovery, coronary care units, and has a total of 200 ICU beds. Patient information was anonymized by replacing the in-hospital patient ID with a surrogate key and shifting time-related information, such as birth date and chart input time, by randomly chosen periods before the analysis. The study was approved by the institutional review board of Severance Hospital, Yonsei University Health System, Seoul, Korea (IRB 4-2017-0939) and Ilsan Hospital (NHIMC 2018-06-004-001). All methods were performed following the Transparent Reporting of a Multivariable Prediction Model for Individual Prognosis or Diagnosis (TRIPOD) guidelines.

### Model Development

We developed prediction models for 3 events: mortality, sepsis, and acute kidney injury (AKI). These are considered major events in the ICU, and prediction of and interventions for these events will help change the clinical course of the patients. The model used 19 features: 6 vital signals, 11 laboratory tests, the Glasgow Coma Score (GCS), and age (see Multimedia Appendix 1). Two of the authors, who are intensive care specialists (KSC and CH), selected features that are widely and routinely used in general ICUs. We excluded any patients who were under the age of 18 years, who did not have at least one valid record with 1 of 5 vital signs (ie, pulse rate, systolic blood pressure, diastolic blood pressure, respiratory rate, and body temperature), and whose event time was after their ICU stay.

The events were identified by the following working definitions. *Mortality* was defined as an in-ICU death recorded in the EMR. According to the clinical surveillance definition [16], *sepsis* was defined as patients who had at least one concurrent acute organ dysfunction. Sepsis was indicated by the initiation of vasopressors or mechanical ventilation; elevated lactate level; or significant changes in the baseline creatinine level, bilirubin level, or platelet count within the 48 hours before or 24 hours after suspected serious infection. *Suspected serious infections* were defined by blood culture and sustained administration of new antibiotics. AKI, according to the Kidney Disease: Improving Global Outcomes (KDIGO) clinical practice guideline [17], was defined as follows: increase in serum creatinine level by 0.3 mg/dL within 48 hours, increase in serum creatinine level to 1.5 times the baseline level that was known or presumed to have occurred within the prior 7 days, or a decrease of 0.5 mL·kg^-1^·h^-1^ in the urine volume for 6 hours. The onset time of the AKI defined the time point at which the creatinine level was elevated.

**Figure 1:**
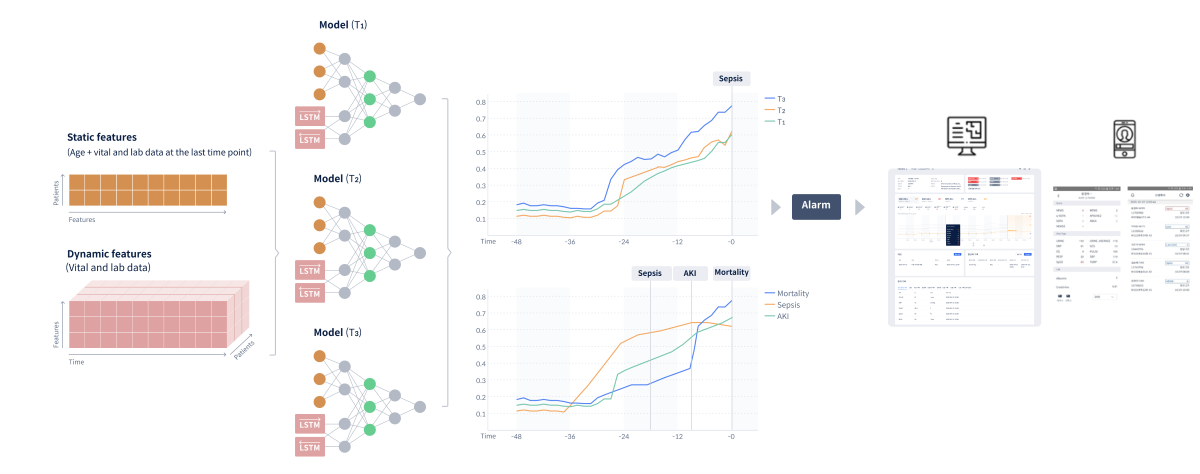
Overview of the model structure. AKI: acute kidney injury; T1: near future; T2: mid-term future; T3: distant future.

Each prediction model was based on bidirectional long short-term memory (biLSTM) and designed as a binary classification model, which answers *yes* or *no* questions. The model used 2 types of data: (1) a dynamic feature, which is time-series data, and (2) a static feature. The sampling frequency of the dynamic feature was 1 hour. We used biLSTM for dynamic features and fully connected layers for static features. We connected the outputs from LSTM and fully connected layers and used them as an input for classification layers (Figure 1). The biLSTM layer has 20 hidden nodes. To train, we use Adam optimizer, a learning rate of 0.001, a batch size of 32, and maximum epochs of 300 (see Multimedia Appendix 2 for details). Additionally, to consider the time interval in which future events will occur, we set 3 future time points: T_1_ (near future), T_2_ (mid-term future), and T_3_ (distant future). Considering the clinical circumstances and shift schedule of medical staff, each event has different time points: mortality and AKI were predicted 3, 6, and 12 hours in advance, and sepsis was predicted 2, 4, and 6 hours in advance. For the model predicting the event within T_i_ (i Î {1, 2, 3}), we preprocessed the data as positive and negative instances. Specifically, we randomly chose some of the time points within T_i_ from the event onset for positive instances and some of the time points within T_i_ from randomly chosen time points for negative instances. After selecting the prediction times, we collected input features from the admission time to the prediction time. Since EMR data have missing data, to impute the missing data, we applied the carry-forward method if valid data existed before the missing time point. If there were no valid data before the missing time point, but valid data existed after the missing time point, we filled the missing value with normal values of the features. To reduce overfitting, we used L2 regularization to the weights of each layer and stopped the model early when the performance of the model for validation set did not improve 60 epochs in a row after the 100th epoch while training each model. To correct the imbalance in outcomes, we used balanced minibatch training.

### Performance Measurements

We compared the model performance with other widely used scores and models. Model performance for mortality was compared to that of the APACHE-II and Sequential Organ Failure Assessment (SOFA) scores, and model performance for sepsis prediction was compared to that for the SOFA score. Although these scores are not gold standards for predicting events, the physician’s decisions have been based on these scores. Therefore, we compared our models with these scores, as in previous studies [18-20]. Additionally, we compared our model with other popular machine learning models (eg, logistic regression and XGBoost) (see Multimedia Appendix 2 for details). However, there are no gold standard scores for AKI; therefore, we compared the model only with other machine learning models for AKI events. The prediction performance of the individual models was measured as the area under the receiver operating characteristic curve (AUROC), area under the precision-recall curve (AUPRC), specificity, and *F*_1_ score with a fixed sensitivity of 0.85, as considered in a previous study [21].

### Validation

The model was validated in two ways: First, 5-fold cross-validation was performed using the development cohort—the standard for evaluation of a machine learning algorithm. Then, the model was externally validated in the independent validation cohort. The validation cohort included patients who were admitted to the ICU of the National Health Insurance Corporation Ilsan Hospital, a secondary hospital run by the national insurance company, between January and December 2017.

### Error and Delayed Input Experiment

The model robustness against entry error and delayed inputs was compared with the two machine learning models by measuring how much the AUROC and SD values were affected by adding noise. To test the robustness to error input, we added Gaussian noise at normalized features with specific ranges (ie, 1/1000, 1/200, 1/100, 1/20, 1/10, 1/2, and one of each feature scale) to randomly chosen data for two vital signs within 10% of the time sequence. Next, to compare with other machine learning models, we added noise on two randomly chosen vital signs. Additionally, we tested the robustness to the delayed input. To make delayed input errors, we deleted the data within specific hours (ie, 0-10 hours) for two randomly chosen vital signs; then, the deleted data were imputed with the carry-forward method.

All analyses were performed using Python (version 3.6.7) [22], and the model was built using the TensorFlow 1.14 [23] deep learning framework.

### Data Availability

The datasets generated during and/or analyzed in this study are not publicly available owing to hospital regulations for electronic medical data but can be made available from the corresponding author upon reasonable request.

## Results

### Demographic Characteristics

A total of 21,732 and 2487 patients were included in the development and validation cohorts, of which 57.13% (n=12,416) and 56.49% (n=1405) were male participants, respectively. The mean participant age was 60.97 (SD 15.2) and 69.05 (SD 14.13) years in the development and validation cohorts, respectively. The prevalence of mortality, sepsis, and AKI was 783 (3.6%), 679 (3.12%), and 1978 (9.15%) in the development cohort and 209 (8.4%), 243 (9.77%), and 287 (11.54%) in the validation cohort Table 1.

**Table 1:** Demographic characteristics of the study cohorts.

Characteristic		Development cohort (n=21,732)	Validation cohort (n=2487)
Patients, n (%)		20,053 (92.27)	2362 (94.97)
Age in years, mean (SD)		60.97 (15.2)	69.05 (14.13)
Sex, male, n (%)		12,416 (57.13)	1405 (56.49)
Death, n (%)		783 (3.6)	209 (8.40)
Sepsis, n (%)		679 (3.12)	243 (9.77)
Acute kidney injury, n (%)		1,978 (9.1)	287 (11.54)
Length of ICU^a^ stay (days), mean (SD)		3.23 (19.15)	2.99 (3.65)
APACHE II^b^ score, mean (SD)		11.57 (5.04)	16.21 (7.25)
SOFA^c^ score, mean (SD)		3.66 (3.01)	4.11 (1.04)
**ICU admission^d^**			
	MICU^e^	3138 (14.44)	606 (24.37)
	SICU^f^	4604 (21.19)	1141 (45.88)
	CCU^g^	5172 (23.79)	740 (29.75)
	HICU^h^	3335 (15.35)	—^i^
	NCU^j^	5483 (25.23)	—

^a^ICU: intensive care unit.

^b^APACHE: Acute Physiology and Chronic Health Evaluation.

^c^SOFA: Sequential Organ Failure Assessment.

^d^Patient could have multiple admissions to the ICU; the sum of the types of ICU admissions exceeds 100%.

^e^MICU: medical intensive care unit.

^f^SICU: surgical intensive care unit

^g^CCU: critical care unit.

^h^HICU: high intensity care unit.

^i^Not available.

^j^NCU: neonatal care unit.

### Model performance

**Figure 2:**
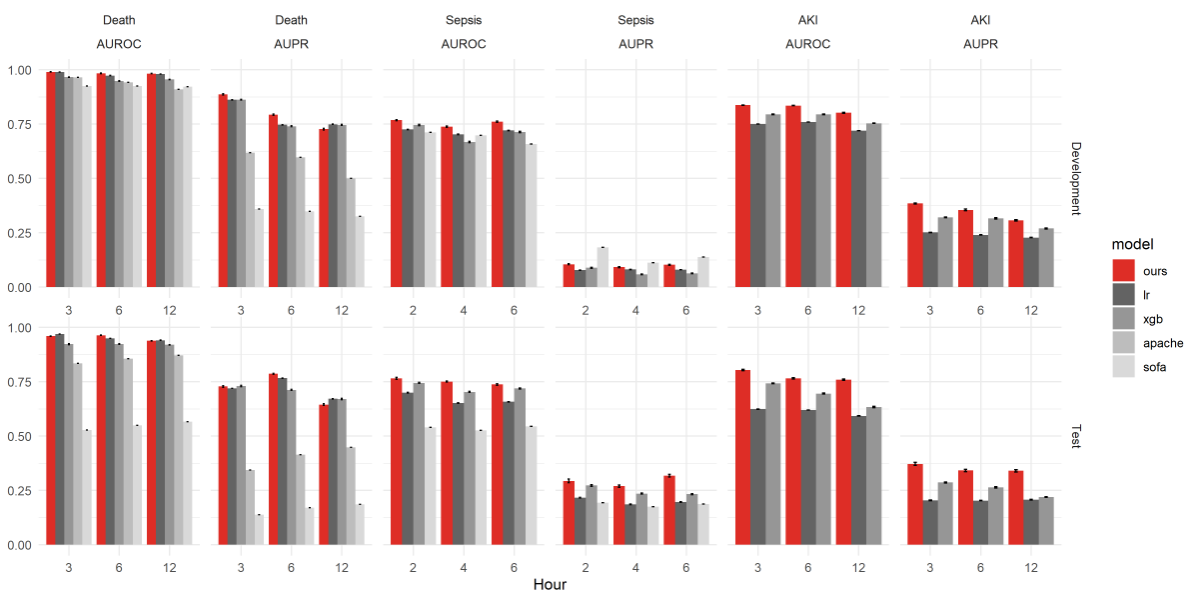
AUROC and AUPR values of each model at each prediction hour. APACHE: Acute Physiology And Chronic Health Evaluation; area under the receiver operating characteristic curve; AUPR: area under the precision-recall curve; SOFA: Sequential Organ Failure Assessment; xgb: XGBoost.

For the development cohort, the AUROC values of the death prediction model 3, 6 and 12 hours in advance were 0.990, 0.984, and 0.982, respectively. For the validation cohort, the model achieved AUROC values of 0.960, 0.964, and 0.938 to predict mortality 3, 6, and 12 hours in advance, respectively. The AUPRC values of the death prediction model 3, 6, 12 hours in advance were 0.887, 0.794, and 0.727, respectively, in the development cohort, and 0.728, 0.786, and 0.645, respectively, in the test cohort. The model compared with APACHE-II, SOFA, logistic regression, and XGBoost models. Our model yielded a higher AUROC and AUPRC value than the other models, except a few points. Moreover, the AUROC values of sepsis prediction models 2, 4, and 6 hours in advance were 0.768, 0.739, and 0.761, respectively, in the development cohort and 0.766, 0.751, and 0.738, respectively, in the test cohort. The AUPRC values of sepsis prediction models 2, 4, and 6 hours in advance were 0.105, 0.092, and 0.103, respectively, in the development cohort and 0.294, 0.270, and 0.318, respectively, in the test cohort. These performances were significantly higher than those using the SOFA score (the gold standard medical score), logistic regression, and XGBoost models, except AUPRC values in the development cohort. Although the AUROC values of our models were higher than SOFA scores, AUPRC values were lower than SOFA scores. Finally, the AUROCs of the AKI prediction model 3, 6, and 12 hours in advance were 0.838, 0.836, and 0.802, respectively, in the development cohort and 0.804, 0.766, and 0.760, respectively, in the test cohort. The AUPRC values of AKI prediction model were 0.385, 0.356, and 0.307, respectively, in the development cohort and 0.372, 0.342, and 0.340, respectively, in the test cohort; these values were higher than those using the other two machine learning models (logistic regression and XGBoost; see Figure 2 and Multimedia Appendices 3 and 4).

### Sensitivity to Error and Delayed Input

**Figure 3:**
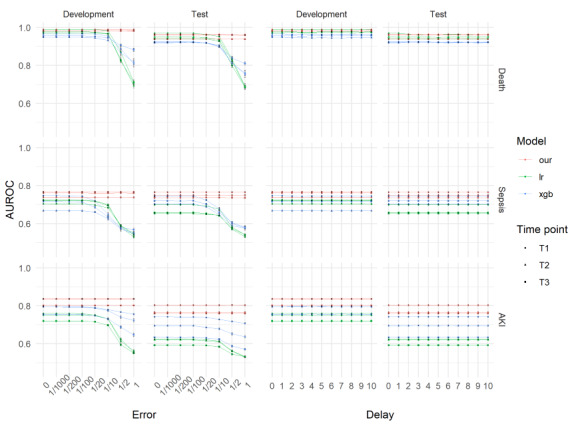
Changes in AUROC when data errors and delayed inputs were simulated. AKI: acute kidney injury; AUROC: area under the receiver operating characteristic curve; LR: logistic regression; T1: near future; T2: mid-term future; T3: distant future; XGB: XGBoost.

The individual models were evaluated by adding data errors as noise. AUROCs of all models except our proposed model were decreased by increasing the added noise. For example, in the mortality prediction model, when adding Gaussian noise with a feature range, the AUROC of our model dropped to 0.0004 (SD 0.002), whereas it was 0.270 (SD 0.0530) for the logistic regression model, and 0.0732 (SD 0.0442) for the XGBoost model, respectively. Other models show similar results. However, in the delayed input experiments, the mean differences in the AUROC between the original and delayed input data were almost 0 in the validation cohort (see Figure 3 and Multimedia Appendix 5).

**Figure 4:**
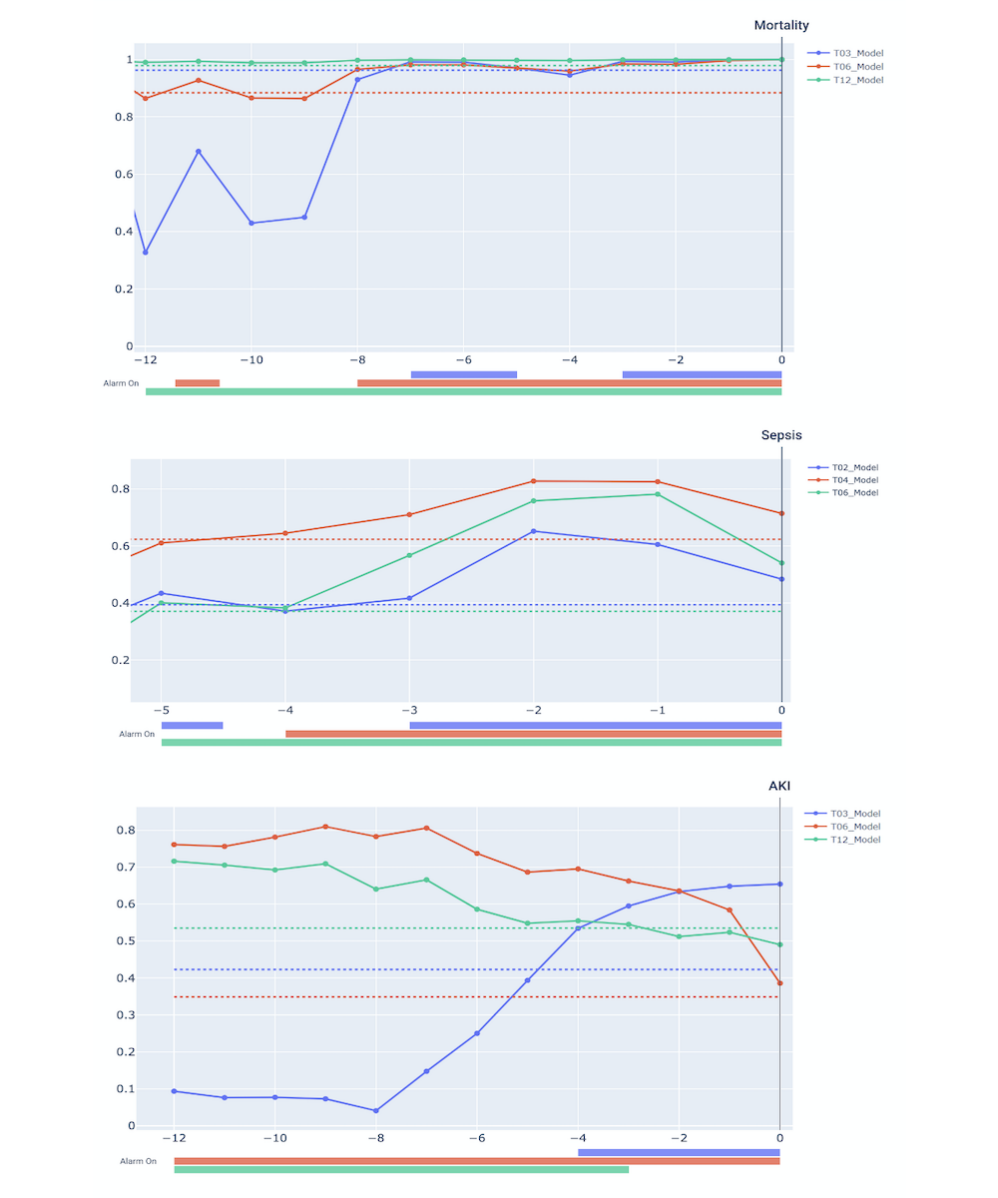
An illustrative example of the prediction of the models. The solid line indicates each model’s score. The dotted line indicates the threshold of each model which set by a sensitivity of 0.85 in (A) Mortality (B) Sepsis (C) AKI. AKI: acute kidney injury.

As shown in Figure 4, each graph shows how each model works. In the mortality prediction model, 12 hours before the event, the alarm is turned on with only the 12-hour model. As the event nears its time, the alarm is turned on with the 6-hour and 3-hour models, sequentially. Other events show similar results. Because each event model predicts different time windows, the models’ prediction can overcome temporal skewness, although there were slight time differences between actual events and predictions.

### Deployment

**Figure 5:**
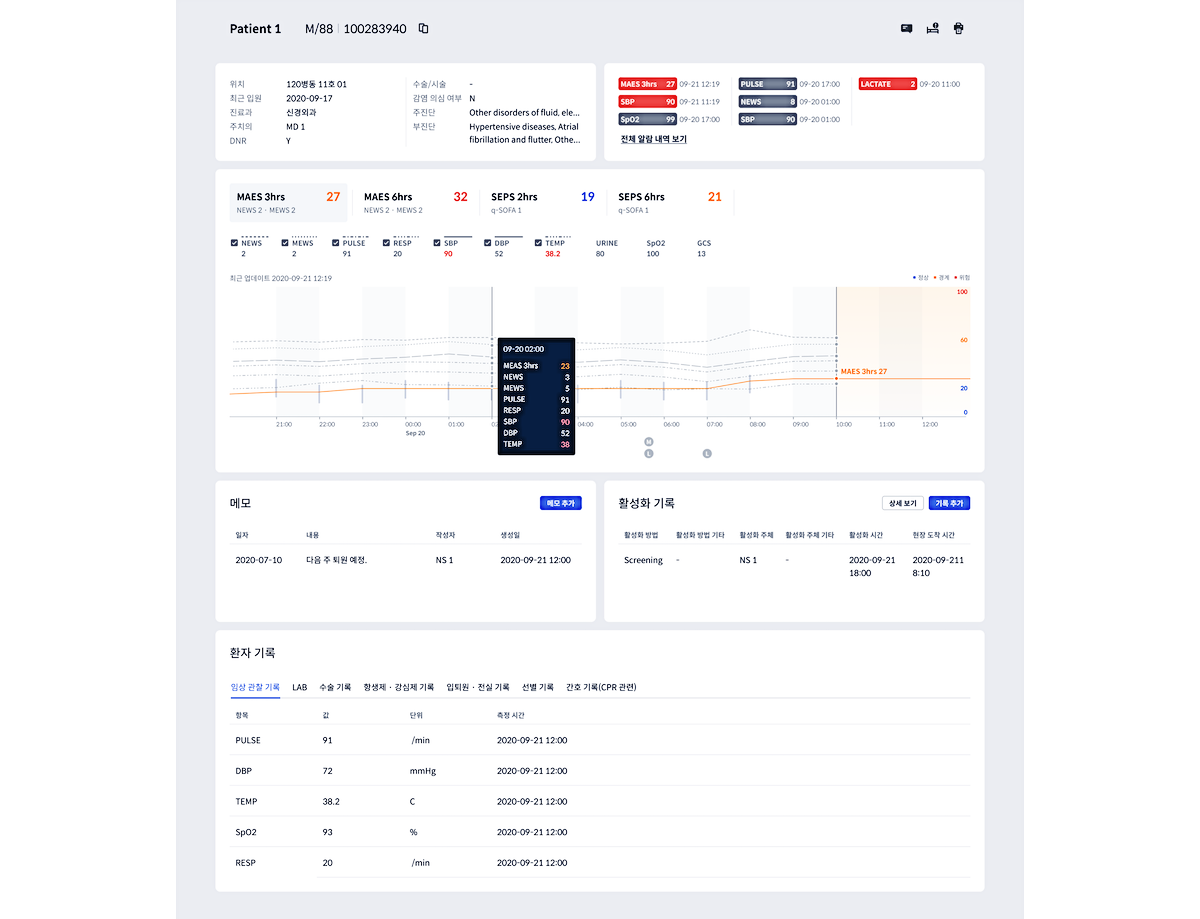
Screenshot of the application on which the model was deployed.

These models have been implemented in tertiary and secondary hospitals in Korea. Figure 5 shows a screenshot of the application used to deploy the models.

## Discussion

### Principal Findings

This study demonstrated the prediction models for events in the ICU that consider not only whether the event occurs but also in which time intervals it would occur. By considering all three models that predict the event at different time intervals, physicians can infer *when* the event would occur. Additionally, the robustness of the model was tested by simulated data errors and delayed input. All models showed similar robustness to delayed input; however, only our proposed model was deemed robust to input errors.

The labeling of the outcomes events is one of the most important things in supervised learning, such as these models. Mortality was defined by the EMR-recorded mortality data. However, for sepsis, according to the Sepsis-3 definition [24], the time point at when the infection was suspected, and organ failure began needs to be known. To overcome this issue, Rhee et al [16] proposed a definition of sepsis for clinical surveillance. Nemati et al [21] defined sepsis similarly except for some time intervals because all the definitions were based on the Sepsis-3 definition. The AKI definition depends on serum creatinine levels. In addition to mortality, since AKI and sepsis were defined by a laboratory test, the event label could be incorrect. This point could make the performance of the two models poorer than that of the mortality prediction model.

Because of this working definition, there was a difference in sepsis prevalence in the two cohorts: the mortality rate was 3.12% and 10.04%, and the sepsis prevalence was 3.12% and 11.17% in the development and validation cohorts, respectively. This is probably because the surgical ICU patients comprised a larger proportion in the development cohort than in the validation cohort. This resulted in the APACHE and SOFA scores of the validation cohort being higher than those of the development cohort.

Many studies have attempted to predict events in the ICU. For instance, Hyland et al [9] developed a model to predict circulatory failure in the ICU. Additionally, circulatory failure in the ICU was assessed using a gradient boosting method with the Shapley Additive Explanations (SHAP) value. The model calculates scores every 5 minutes to predict the risk of circulatory failure within the next 8 hours, and it has an AUROC of 0.90. However, because the model was developed as a within-setting model, it is not clear how long it will take for the event to occur. The model only predicts whether the event will occur within 8 hours, even though the event could occur after only 1 hour. Meyer et al [10] predicted mortality, bleeding, and the need for renal replacement therapy 24 hours after cardiothoracic surgery; the AUROCs for these events were 0.87, 0.95, and 0.96, respectively. Even though the model predicted real-time events, the outcome was fixed-time events. Nemati et al predicted sepsis in the ICU using 65 features, including EMR and high-resolution bedside monitoring data; their model yielded AUROCs of 0.82, 0.81, 0.80, and 0.79 for predicting sepsis 4, 6, 8, and 12 hours in advance, respectively. The model was based on the Weibull-Cox regression model, considering within-setting timepoints. To overcome the temporal skewness of the model, Kim et al [11] developed a model that predicts the time point of in-hospital cardiac arrest using a character-level gated recurrent unit with a Weibull distribution. They assumed that the temporal skewness conformed to the Weibull distribution and then predicted the time point at which the distribution indicated the maximum value. Our data also showed temporal skewness of the positive events. When plotted the event-prediction time with each group, most of the predicted true event was found to occur near the real event occurrences (Multimedia Appendix 6). This phenomenon can be shown in other time prediction models. The temporal skewness is important when the model is applied in the real world. When physicians receive an alarm from the model, the working time—that is, the time between the alarm alert and the real event—should be enough to intervene disease progression.

The mortality and AKI predict model showed that the nearer the prediction time was to the event time, the higher the AUROC value was. However, the analyses pertaining to sepsis events, showed the 6 hours in advance prediction model worked better than the 2 hours in advance prediction model. This might be because the definition of sepsis is more subjective than that of other events.

Most robustness assessments of previous models have focused on generalization to any data input. For example, weight decay [25] and the early stopping method [26] are well-known approaches that make the model more robust. However, in this study, we focused on robustness to error and delayed input. All the models showed robustness to the delayed input. This may be because the carry-forward method (used to impute the deleted data) resulted in the delayed input data not being considerably different from the original data, unlike the noise-add experiment. However, the error input experiment showed that our models were more robust than other models (Figure 3). Although we randomly selected two vital signs in the error input experiment, we performed the sensitive analysis by selecting specific pairs of vital signs and adding noise only to those pairs. The mean differences between the original model and the noise added model considered on a scale of 1 were less than 0.003. The performance was still similar such that vital signs were selected, and noises were added (Multimedia appendix 7). Moreover, unlike the time-series model that requires values from time windows, the non–time-series model needs one abstracted value. It seems that making values abstract can lead to higher robustness than the time-series model. However, the non–time series models yielded lower AUROC values than those of our models, except the sepsis prediction model with test dataset (Multimedia Appendix 8). This finding suggested that the time-series model yielded a higher performance and was more robust to the error and delayed input (see Figure 3) than the non–time series models. This can be explained by the fact that the time-series model learned from all the time-series features rather than one time-series representative value.

To the best of our knowledge, this is the first attempt to evaluate the robustness of the model against delayed input and input error. There was no metric for the robustness of an error and delayed input. Thus, the AUROC variation—that is, the mean difference—was used to evaluate robustness when noise was added, or the input was delayed.

### Limitations

There are some limitations to our study. First, we could not consider the correlation between each event. For example, both mortality and AKI can be caused by sepsis. However, in this model, each event was considered an independent outcome. Further research should be performed to predict these correlated outcomes. Second, we evaluated input error and delayed input by adding simulated noise to retrospective data. In addition, the model works in the real world. To evaluate these points, a prospective study should be performed. Third, the input features were selected manually; however, these few variables are commonly used in ICUs worldwide to predict patient outcomes. According to survey on sepsis prediction [27], our features have been included in other models. Additionally, other clinical complications or adverse events should be expanded in future studies.

### Conclusions

In this study, we developed an outcome prediction model for real-world applications. We considered not only performance but also the robustness of the model to temporal skewness and input delays and errors. By considering temporal skewness, physicians can more effectively intervene in disease progression. Additionally, since the models are robust to delayed input and input error, physicians can trust this model more than those that are not as robust.

## Appendix

Multimedia Appendix 1 The input features of the model.

Multimedia Appendix 2 Logistic regression, XGBoost, and LSTM hyperparameters. LSTM: long short-term memory.

Multimedia Appendix 3 The results of AUROC and AUPRC of each model and prediction hour. AUPRC: area under the precision-recall curve;  AUROC: area under the receiver operating characteristic curve.

Multimedia Appendix 4 The comparison among AUROC values by each prediction hour in our models. AUROC: area under the receiver operating characteristic curve.

Multimedia Appendix 5 Mean and standard deviation of AUROC values in adding noise and input delayed experiments. AUROC: area under the receiver operating characteristic curve.

Multimedia Appendix 6 Distribution of true positive samples with event to prediction temporal gap.

Multimedia Appendix 7 Error input experiment with two fixed selected vital signs.

Multimedia Appendix 8 The AUROC values of our model and non-time-series model which inputted several representative values, such as highest, median, and lowest value of all time windows. AUROC: area under the receiver operating characteristic curve.

## Abbreviations

AKI: acute kidney injury
APACHE: Acute Physiology And Chronic Health Evaluation
AUPRC: area under the precision-recall curve
AUROC: area under the receiver operating characteristic curve
EMR: electronic medical record
GCS: Glasgow Coma Score
ICU: intensive care unit
IITP: Institute for Information & Communication Technology Planning & Evaluation
IoMT: internet of medical things
MPM: Mortality Probability Model
MSIT: Ministry of Science and Information and Communications Technology
SAPS: Simplified Acute Physiology Score
SOFA: Sequential Organ Failure Assessment
TRIPOD: Transparent Reporting of a Multivariable Prediction Model for Individual Prognosis or Diagnosis
