# Supplementary material for: Event Prediction Model Considering Time and Input Error Using Electronic Medical Records in the Intensive Care Unit: Retrospective Study
Source: JMIR Med Inform. 2021 Nov 4;9(11):e26426. doi: 10.2196/26426 (PMC8603167; doi:10.2196/26426)
Supplement: Multimedia Appendix 1 [file medinform_v9i11e26426_app1.docx]

**Multimedia Appendix 1.** **The input features of the model**

| **Category** | **Input features** |
| --- | --- |
| Vital signs | Heart rate, Respiration Rate, Temperature, SBP, MBP, DBP, SpO2 |
| Laboratory test | Bilirubin, Lactate, PH, Sodium, Potassium, Creatinine, Hematocrit, WBCHCO3, Platelet |
| Neurologic test | the Glasgow coma score |
| Demographic | Age |
