# Supplementary material for: Event Prediction Model Considering Time and Input Error Using Electronic Medical Records in the Intensive Care Unit: Retrospective Study
Source: JMIR Med Inform. 2021 Nov 4;9(11):e26426. doi: 10.2196/26426 (PMC8603167; doi:10.2196/26426)
Supplement: Multimedia Appendix 2 [file medinform_v9i11e26426_app2.docx]

**Multimedia Appendix 2. Logistic regression, XGBoost, and LSTM hyperparameters**

Logistic regression Hyperparameters

| Penalty | l2 |
| --- | --- |
| Optimization | lbfgs |
| Max Iteration | 100 |
| Regularization strength | 1.0 |
| Input Features | Age  Lastest time-variant value |

XGBoost hyperparameters

| Max depth | 5 |
| --- | --- |
| Learning rate | 0.01 |
| Gamma | 0 |
| Booster | gbtree |
| Min child Weight | 1 |
| Objective | Binary logistic regression |
| Input Features | Age  Lastest time-variant value |

LSTM hyperparameters

| Number of biLSTM layers | 1 |
| --- | --- |
| Sampling frequency | 1 Hour |
| Optimizer | Adam |
| Learning rate | 0.001 |
| Batch size | 32 |
| Maximum epochs | 300 |
| Number of hidden nodes in LSTM | 20 |
| Input Features | Age  All time-variant values |
