# Supplementary material for: Event Prediction Model Considering Time and Input Error Using Electronic Medical Records in the Intensive Care Unit: Retrospective Study
Source: JMIR Med Inform. 2021 Nov 4;9(11):e26426. doi: 10.2196/26426 (PMC8603167; doi:10.2196/26426)
Supplement: Multimedia Appendix 3 [file medinform_v9i11e26426_app3.docx]

**Multimedia Appendix 3. The results of AUROCs and AUPRCs of each model and prediction hour.**

| Hospital | Target | Hour | model | measured | value | error |
| --- | --- | --- | --- | --- | --- | --- |
| Development | Death | 12 | ours | AUROC | 0.982 | 0.001 |
| Development | Death | 12 | lr | AUROC | 0.980 | 0.000 |
| Development | Death | 12 | xgb | AUROC | 0.956 | 0.001 |
| Development | Death | 12 | apache | AUROC | 0.910 | 0.000 |
| Development | Death | 12 | sofa | AUROC | 0.922 | 0.000 |
| Development | Death | 12 | ours | AUPR | 0.727 | 0.004 |
| Development | Death | 12 | lr | AUPR | 0.750 | 0.000 |
| Development | Death | 12 | xgb | AUPR | 0.746 | 0.003 |
| Development | Death | 12 | apache | AUPR | 0.501 | 0.000 |
| Development | Death | 12 | sofa | AUPR | 0.325 | 0.000 |
| Development | Death | 6 | ours | AUROC | 0.984 | 0.001 |
| Development | Death | 6 | lr | AUROC | 0.973 | 0.001 |
| Development | Death | 6 | xgb | AUROC | 0.948 | 0.001 |
| Development | Death | 6 | apache | AUROC | 0.943 | 0.000 |
| Development | Death | 6 | sofa | AUROC | 0.925 | 0.000 |
| Development | Death | 6 | ours | AUPR | 0.794 | 0.003 |
| Development | Death | 6 | lr | AUPR | 0.747 | 0.001 |
| Development | Death | 6 | xgb | AUPR | 0.740 | 0.002 |
| Development | Death | 6 | apache | AUPR | 0.597 | 0.000 |
| Development | Death | 6 | sofa | AUPR | 0.349 | 0.000 |
| Development | Death | 3 | ours | AUROC | 0.990 | 0.000 |
| Development | Death | 3 | lr | AUROC | 0.990 | 0.000 |
| Development | Death | 3 | xgb | AUROC | 0.966 | 0.001 |
| Development | Death | 3 | apache | AUROC | 0.965 | 0.000 |
| Development | Death | 3 | sofa | AUROC | 0.925 | 0.000 |
| Development | Death | 3 | ours | AUPR | 0.887 | 0.003 |
| Development | Death | 3 | lr | AUPR | 0.862 | 0.001 |
| Development | Death | 3 | xgb | AUPR | 0.862 | 0.001 |
| Development | Death | 3 | apache | AUPR | 0.619 | 0.000 |
| Development | Death | 3 | sofa | AUPR | 0.359 | 0.000 |
| Development | Sepsis | 6 | ours | AUROC | 0.761 | 0.003 |
| Development | Sepsis | 6 | lr | AUROC | 0.721 | 0.001 |
| Development | Sepsis | 6 | xgb | AUROC | 0.714 | 0.003 |
| Development | Sepsis | 6 | sofa | AUROC | 0.659 | 0.000 |
| Development | Sepsis | 6 | ours | AUPR | 0.103 | 0.003 |
| Development | Sepsis | 6 | lr | AUPR | 0.080 | 0.000 |
| Development | Sepsis | 6 | xgb | AUPR | 0.063 | 0.001 |
| Development | Sepsis | 6 | sofa | AUPR | 0.139 | 0.000 |
| Development | Sepsis | 4 | ours | AUROC | 0.739 | 0.003 |
| Development | Sepsis | 4 | lr | AUROC | 0.703 | 0.001 |
| Development | Sepsis | 4 | xgb | AUROC | 0.668 | 0.003 |
| Development | Sepsis | 4 | sofa | AUROC | 0.698 | 0.000 |
| Development | Sepsis | 4 | ours | AUPR | 0.092 | 0.002 |
| Development | Sepsis | 4 | lr | AUPR | 0.082 | 0.001 |
| Development | Sepsis | 4 | xgb | AUPR | 0.059 | 0.001 |
| Development | Sepsis | 4 | sofa | AUPR | 0.113 | 0.000 |
| Development | Sepsis | 2 | ours | AUROC | 0.768 | 0.003 |
| Development | Sepsis | 2 | lr | AUROC | 0.726 | 0.001 |
| Development | Sepsis | 2 | xgb | AUROC | 0.746 | 0.003 |
| Development | Sepsis | 2 | sofa | AUROC | 0.713 | 0.000 |
| Development | Sepsis | 2 | ours | AUPR | 0.105 | 0.002 |
| Development | Sepsis | 2 | lr | AUPR | 0.078 | 0.001 |
| Development | Sepsis | 2 | xgb | AUPR | 0.089 | 0.002 |
| Development | Sepsis | 2 | sofa | AUPR | 0.183 | 0.000 |
| Development | AKI | 12 | ours | AUROC | 0.802 | 0.002 |
| Development | AKI | 12 | lr | AUROC | 0.720 | 0.000 |
| Development | AKI | 12 | xgb | AUROC | 0.754 | 0.001 |
| Development | AKI | 12 | ours | AUPR | 0.307 | 0.003 |
| Development | AKI | 12 | lr | AUPR | 0.228 | 0.001 |
| Development | AKI | 12 | xgb | AUPR | 0.270 | 0.002 |
| Development | AKI | 6 | ours | AUROC | 0.836 | 0.001 |
| Development | AKI | 6 | lr | AUROC | 0.760 | 0.000 |
| Development | AKI | 6 | xgb | AUROC | 0.796 | 0.001 |
| Development | AKI | 6 | ours | AUPR | 0.356 | 0.004 |
| Development | AKI | 6 | lr | AUPR | 0.240 | 0.000 |
| Development | AKI | 6 | xgb | AUPR | 0.317 | 0.003 |
| Development | AKI | 3 | ours | AUROC | 0.838 | 0.001 |
| Development | AKI | 3 | lr | AUROC | 0.750 | 0.000 |
| Development | AKI | 3 | xgb | AUROC | 0.795 | 0.001 |
| Development | AKI | 3 | ours | AUPR | 0.385 | 0.003 |
| Development | AKI | 3 | lr | AUPR | 0.252 | 0.001 |
| Development | AKI | 3 | xgb | AUPR | 0.321 | 0.002 |
| Test | Death | 12 | ours | AUROC | 0.938 | 0.001 |
| Test | Death | 12 | lr | AUROC | 0.941 | 0.001 |
| Test | Death | 12 | xgb | AUROC | 0.920 | 0.001 |
| Test | Death | 12 | apache | AUROC | 0.872 | 0.000 |
| Test | Death | 12 | sofa | AUROC | 0.566 | 0.000 |
| Test | Death | 12 | ours | AUPR | 0.645 | 0.004 |
| Test | Death | 12 | lr | AUPR | 0.672 | 0.001 |
| Test | Death | 12 | xgb | AUPR | 0.670 | 0.002 |
| Test | Death | 12 | apache | AUPR | 0.448 | 0.000 |
| Test | Death | 12 | sofa | AUPR | 0.185 | 0.000 |
| Test | Death | 6 | ours | AUROC | 0.964 | 0.001 |
| Test | Death | 6 | lr | AUROC | 0.950 | 0.000 |
| Test | Death | 6 | xgb | AUROC | 0.924 | 0.002 |
| Test | Death | 6 | apache | AUROC | 0.856 | 0.000 |
| Test | Death | 6 | sofa | AUROC | 0.550 | 0.000 |
| Test | Death | 6 | ours | AUPR | 0.786 | 0.002 |
| Test | Death | 6 | lr | AUPR | 0.767 | 0.001 |
| Test | Death | 6 | xgb | AUPR | 0.712 | 0.003 |
| Test | Death | 6 | apache | AUPR | 0.414 | 0.000 |
| Test | Death | 6 | sofa | AUPR | 0.170 | 0.000 |
| Test | Death | 3 | ours | AUROC | 0.960 | 0.001 |
| Test | Death | 3 | lr | AUROC | 0.970 | 0.000 |
| Test | Death | 3 | xgb | AUROC | 0.923 | 0.002 |
| Test | Death | 3 | apache | AUROC | 0.834 | 0.000 |
| Test | Death | 3 | sofa | AUROC | 0.528 | 0.000 |
| Test | Death | 3 | ours | AUPR | 0.728 | 0.004 |
| Test | Death | 3 | lr | AUPR | 0.720 | 0.000 |
| Test | Death | 3 | xgb | AUPR | 0.731 | 0.003 |
| Test | Death | 3 | apache | AUPR | 0.343 | 0.000 |
| Test | Death | 3 | sofa | AUPR | 0.138 | 0.000 |
| Test | Sepsis | 6 | ours | AUROC | 0.738 | 0.004 |
| Test | Sepsis | 6 | lr | AUROC | 0.658 | 0.001 |
| Test | Sepsis | 6 | xgb | AUROC | 0.719 | 0.002 |
| Test | Sepsis | 6 | sofa | AUROC | 0.545 | 0.000 |
| Test | Sepsis | 6 | ours | AUPR | 0.318 | 0.006 |
| Test | Sepsis | 6 | lr | AUPR | 0.197 | 0.001 |
| Test | Sepsis | 6 | xgb | AUPR | 0.233 | 0.003 |
| Test | Sepsis | 6 | sofa | AUPR | 0.187 | 0.000 |
| Test | Sepsis | 4 | ours | AUROC | 0.751 | 0.003 |
| Test | Sepsis | 4 | lr | AUROC | 0.652 | 0.001 |
| Test | Sepsis | 4 | xgb | AUROC | 0.703 | 0.002 |
| Test | Sepsis | 4 | sofa | AUROC | 0.527 | 0.000 |
| Test | Sepsis | 4 | ours | AUPR | 0.270 | 0.006 |
| Test | Sepsis | 4 | lr | AUPR | 0.186 | 0.002 |
| Test | Sepsis | 4 | xgb | AUPR | 0.235 | 0.003 |
| Test | Sepsis | 4 | sofa | AUPR | 0.175 | 0.000 |
| Test | Sepsis | 2 | ours | AUROC | 0.766 | 0.004 |
| Test | Sepsis | 2 | lr | AUROC | 0.699 | 0.001 |
| Test | Sepsis | 2 | xgb | AUROC | 0.745 | 0.002 |
| Test | Sepsis | 2 | sofa | AUROC | 0.540 | 0.000 |
| Test | Sepsis | 2 | ours | AUPR | 0.294 | 0.008 |
| Test | Sepsis | 2 | lr | AUPR | 0.216 | 0.001 |
| Test | Sepsis | 2 | xgb | AUPR | 0.273 | 0.004 |
| Test | Sepsis | 2 | sofa | AUPR | 0.193 | 0.000 |
| Test | AKI | 12 | ours | AUROC | 0.760 | 0.003 |
| Test | AKI | 12 | lr | AUROC | 0.592 | 0.001 |
| Test | AKI | 12 | xgb | AUROC | 0.634 | 0.003 |
| Test | AKI | 12 | ours | AUPR | 0.340 | 0.006 |
| Test | AKI | 12 | lr | AUPR | 0.207 | 0.001 |
| Test | AKI | 12 | xgb | AUPR | 0.220 | 0.002 |
| Test | AKI | 6 | ours | AUROC | 0.766 | 0.003 |
| Test | AKI | 6 | lr | AUROC | 0.620 | 0.000 |
| Test | AKI | 6 | xgb | AUROC | 0.696 | 0.003 |
| Test | AKI | 6 | ours | AUPR | 0.342 | 0.005 |
| Test | AKI | 6 | lr | AUPR | 0.203 | 0.001 |
| Test | AKI | 6 | xgb | AUPR | 0.264 | 0.003 |
| Test | AKI | 3 | ours | AUROC | 0.804 | 0.003 |
| Test | AKI | 3 | lr | AUROC | 0.624 | 0.001 |
| Test | AKI | 3 | xgb | AUROC | 0.742 | 0.002 |
| Test | AKI | 3 | ours | AUPR | 0.372 | 0.006 |
| Test | AKI | 3 | lr | AUPR | 0.204 | 0.001 |
| Test | AKI | 3 | xgb | AUPR | 0.286 | 0.003 |
