# Supplementary material for: Event Prediction Model Considering Time and Input Error Using Electronic Medical Records in the Intensive Care Unit: Retrospective Study
Source: JMIR Med Inform. 2021 Nov 4;9(11):e26426. doi: 10.2196/26426 (PMC8603167; doi:10.2196/26426)
Supplement: Multimedia Appendix 4 [file medinform_v9i11e26426_app4.docx]

**Multimedia Appendix 4. The comparison among AUROCs by each prediction hour**

**in our model**

| Hospital | target | group1 | group2 | statistic | p.adj | signif |
| --- | --- | --- | --- | --- | --- | --- |
| Development | Death | 3 | 6 | 7.141428 | 4.42E-07 | **** |
| Development | Death | 3 | 12 | 8 | 9.48E-08 | **** |
| Development | Death | 6 | 12 | 1.140704 | 0.265 | ns |
| Test | Death | 3 | 6 | -4.34248 | 2.21E-04 | *** |
| Test | Death | 3 | 12 | 17.66667 | 5.91E-15 | **** |
| Test | Death | 6 | 12 | 17.98245 | 5.91E-15 | **** |
| Development | Sepsis | 2 | 4 | 6.762293 | 1.62E-06 | **** |
| Development | Sepsis | 2 | 6 | 1.754533 | 0.092 | ns |
| Development | Sepsis | 4 | 6 | -5.83049 | 1.04E-05 | **** |
| Test | Sepsis | 2 | 4 | 3.278919 | 0.006 | ** |
| Test | Sepsis | 2 | 6 | 5.491252 | 3.60E-05 | **** |
| Test | Sepsis | 4 | 6 | 3.309667 | 0.006 | ** |
| Development | AKI | 3 | 6 | 1.224745 | 0.233 | ns |
| Development | AKI | 3 | 12 | 18.52425 | 3.03E-15 | **** |
| Development | AKI | 6 | 12 | 15.62087 | 8.94E-14 | **** |
| Test | AKI | 3 | 6 | 9.094409 | 6.06E-09 | **** |
| Test | AKI | 3 | 12 | 10.19389 | 1.01E-09 | **** |
| Test | AKI | 6 | 12 | 1.732051 | 0.096 | ns |
