# Supplementary material for: Event Prediction Model Considering Time and Input Error Using Electronic Medical Records in the Intensive Care Unit: Retrospective Study
Source: JMIR Med Inform. 2021 Nov 4;9(11):e26426. doi: 10.2196/26426 (PMC8603167; doi:10.2196/26426)
Supplement: Multimedia Appendix 5 [file medinform_v9i11e26426_app5.docx]

**Multimedia Appendix 5. The mean and standard deviation of AUROCs in adding noise and input delayed experiments**

| **hour** | **hospital** | **target** | **experiment** | **Model** | **mean_AUROC** | **sd_AUROC** |
| --- | --- | --- | --- | --- | --- | --- |
| T1 | Severance | Death | Error | lr | 0.872 | 0.167 |
| T1 | Severance | Death | Error | our | 0.983 | 0.000 |
| T1 | Severance | Death | Error | xgb | 0.884 | 0.100 |
| T1 | Severance | Sepsis | Error | lr | 0.637 | 0.069 |
| T1 | Severance | Sepsis | Error | our | 0.733 | 0.001 |
| T1 | Severance | Sepsis | Error | xgb | 0.618 | 0.062 |
| T1 | Severance | AKI | Error | lr | 0.679 | 0.093 |
| T1 | Severance | AKI | Error | our | 0.771 | 0.002 |
| T1 | Severance | AKI | Error | xgb | 0.726 | 0.042 |
| T1 | Ilsan | Death | Error | lr | 0.863 | 0.128 |
| T1 | Ilsan | Death | Error | our | 0.926 | 0.003 |
| T1 | Ilsan | Death | Error | xgb | 0.905 | 0.052 |
| T1 | Ilsan | Sepsis | Error | lr | 0.627 | 0.059 |
| T1 | Ilsan | Sepsis | Error | our | 0.816 | 0.000 |
| T1 | Ilsan | Sepsis | Error | xgb | 0.677 | 0.025 |
| T1 | Ilsan | AKI | Error | lr | 0.575 | 0.030 |
| T1 | Ilsan | AKI | Error | our | 0.707 | 0.007 |
| T1 | Ilsan | AKI | Error | xgb | 0.582 | 0.011 |
| T2 | Severance | Death | Error | lr | 0.874 | 0.157 |
| T2 | Severance | Death | Error | our | 0.971 | 0.000 |
| T2 | Severance | Death | Error | xgb | 0.893 | 0.075 |
| T2 | Severance | Sepsis | Error | lr | 0.699 | 0.099 |
| T2 | Severance | Sepsis | Error | our | 0.770 | 0.001 |
| T2 | Severance | Sepsis | Error | xgb | 0.682 | 0.099 |
| T2 | Severance | AKI | Error | lr | 0.671 | 0.075 |
| T2 | Severance | AKI | Error | our | 0.773 | 0.006 |
| T2 | Severance | AKI | Error | xgb | 0.704 | 0.064 |
| T2 | Ilsan | Death | Error | lr | 0.915 | 0.105 |
| T2 | Ilsan | Death | Error | our | 0.961 | 0.001 |
| T2 | Ilsan | Death | Error | xgb | 0.921 | 0.010 |
| T2 | Ilsan | Sepsis | Error | lr | 0.666 | 0.078 |
| T2 | Ilsan | Sepsis | Error | our | 0.753 | 0.002 |
| T2 | Ilsan | Sepsis | Error | xgb | 0.685 | 0.051 |
| T2 | Ilsan | AKI | Error | lr | 0.585 | 0.029 |
| T2 | Ilsan | AKI | Error | our | 0.711 | 0.002 |
| T2 | Ilsan | AKI | Error | xgb | 0.609 | 0.022 |
| T3 | Severance | Death | Error | lr | 0.899 | 0.160 |
| T3 | Severance | Death | Error | our | 0.994 | 0.000 |
| T3 | Severance | Death | Error | xgb | 0.916 | 0.097 |
| T3 | Severance | Sepsis | Error | lr | 0.669 | 0.079 |
| T3 | Severance | Sepsis | Error | our | 0.783 | 0.006 |
| T3 | Severance | Sepsis | Error | xgb | 0.695 | 0.064 |
| T3 | Severance | AKI | Error | lr | 0.667 | 0.087 |
| T3 | Severance | AKI | Error | our | 0.790 | 0.009 |
| T3 | Severance | AKI | Error | xgb | 0.722 | 0.039 |
| T3 | Ilsan | Death | Error | lr | 0.907 | 0.113 |
| T3 | Ilsan | Death | Error | our | 0.956 | 0.000 |
| T3 | Ilsan | Death | Error | xgb | 0.887 | 0.061 |
| T3 | Ilsan | Sepsis | Error | lr | 0.640 | 0.071 |
| T3 | Ilsan | Sepsis | Error | our | 0.731 | 0.001 |
| T3 | Ilsan | Sepsis | Error | xgb | 0.671 | 0.024 |
| T3 | Ilsan | AKI | Error | lr | 0.600 | 0.044 |
| T3 | Ilsan | AKI | Error | our | 0.762 | 0.008 |
| T3 | Ilsan | AKI | Error | xgb | 0.695 | 0.024 |
| T1 | Severance | Death | Delay | lr | 0.985 | 0.000 |
| T1 | Severance | Death | Delay | our | 0.997 | 0.000 |
| T1 | Severance | Death | Delay | xgb | 0.975 | 0.000 |
| T1 | Severance | Sepsis | Delay | lr | 0.677 | 0.004 |
| T1 | Severance | Sepsis | Delay | our | 0.734 | 0.000 |
| T1 | Severance | Sepsis | Delay | xgb | 0.673 | 0.004 |
| T1 | Severance | AKI | Delay | lr | 0.741 | 0.001 |
| T1 | Severance | AKI | Delay | our | 0.772 | 0.000 |
| T1 | Severance | AKI | Delay | xgb | 0.757 | 0.001 |
| T1 | Ilsan | Death | Delay | lr | 0.937 | 0.001 |
| T1 | Ilsan | Death | Delay | our | 0.927 | 0.000 |
| T1 | Ilsan | Death | Delay | xgb | 0.931 | 0.000 |
| T1 | Ilsan | Sepsis | Delay | lr | 0.661 | 0.006 |
| T1 | Ilsan | Sepsis | Delay | our | 0.816 | 0.000 |
| T1 | Ilsan | Sepsis | Delay | xgb | 0.693 | 0.002 |
| T1 | Ilsan | AKI | Delay | lr | 0.590 | 0.000 |
| T1 | Ilsan | AKI | Delay | our | 0.711 | 0.000 |
| T1 | Ilsan | AKI | Delay | xgb | 0.591 | 0.001 |
| T2 | Severance | Death | Delay | lr | 0.960 | 0.000 |
| T2 | Severance | Death | Delay | our | 0.986 | 0.000 |
| T2 | Severance | Death | Delay | xgb | 0.957 | 0.000 |
| T2 | Severance | Sepsis | Delay | lr | 0.757 | 0.004 |
| T2 | Severance | Sepsis | Delay | our | 0.769 | 0.000 |
| T2 | Severance | Sepsis | Delay | xgb | 0.769 | 0.002 |
| T2 | Severance | AKI | Delay | lr | 0.722 | 0.000 |
| T2 | Severance | AKI | Delay | our | 0.775 | 0.000 |
| T2 | Severance | AKI | Delay | xgb | 0.753 | 0.000 |
| T2 | Ilsan | Death | Delay | lr | 0.969 | 0.001 |
| T2 | Ilsan | Death | Delay | our | 0.962 | 0.000 |
| T2 | Ilsan | Death | Delay | xgb | 0.929 | 0.002 |
| T2 | Ilsan | Sepsis | Delay | lr | 0.713 | 0.006 |
| T2 | Ilsan | Sepsis | Delay | our | 0.751 | 0.000 |
| T2 | Ilsan | Sepsis | Delay | xgb | 0.730 | 0.008 |
| T2 | Ilsan | AKI | Delay | lr | 0.601 | 0.000 |
| T2 | Ilsan | AKI | Delay | our | 0.710 | 0.000 |
| T2 | Ilsan | AKI | Delay | xgb | 0.618 | 0.000 |
| T3 | Severance | Death | Delay | lr | 0.972 | 0.000 |
| T3 | Severance | Death | Delay | our | 0.986 | 0.000 |
| T3 | Severance | Death | Delay | xgb | 0.932 | 0.001 |
| T3 | Severance | Sepsis | Delay | lr | 0.702 | 0.010 |
| T3 | Severance | Sepsis | Delay | our | 0.786 | 0.000 |
| T3 | Severance | Sepsis | Delay | xgb | 0.737 | 0.011 |
| T3 | Severance | AKI | Delay | lr | 0.724 | 0.000 |
| T3 | Severance | AKI | Delay | our | 0.795 | 0.000 |
| T3 | Severance | AKI | Delay | xgb | 0.750 | 0.000 |
| T3 | Ilsan | Death | Delay | lr | 0.951 | 0.000 |
| T3 | Ilsan | Death | Delay | our | 0.956 | 0.000 |
| T3 | Ilsan | Death | Delay | xgb | 0.912 | 0.000 |
| T3 | Ilsan | Sepsis | Delay | lr | 0.680 | 0.006 |
| T3 | Ilsan | Sepsis | Delay | our | 0.731 | 0.000 |
| T3 | Ilsan | Sepsis | Delay | xgb | 0.685 | 0.002 |
| T3 | Ilsan | AKI | Delay | lr | 0.622 | 0.000 |
| T3 | Ilsan | AKI | Delay | our | 0.766 | 0.000 |
| T3 | Ilsan | AKI | Delay | xgb | 0.708 | 0.000 |
