# Supplementary figures and images for: Event Prediction Model Considering Time and Input Error Using Electronic Medical Records in the Intensive Care Unit: Retrospective Study
Source: JMIR Med Inform. 2021 Nov 4;9(11):e26426. doi: 10.2196/26426 (PMC8603167; doi:10.2196/26426)

**Multimedia appendix 7. The error input experiment with two fixed selected vital signs.**


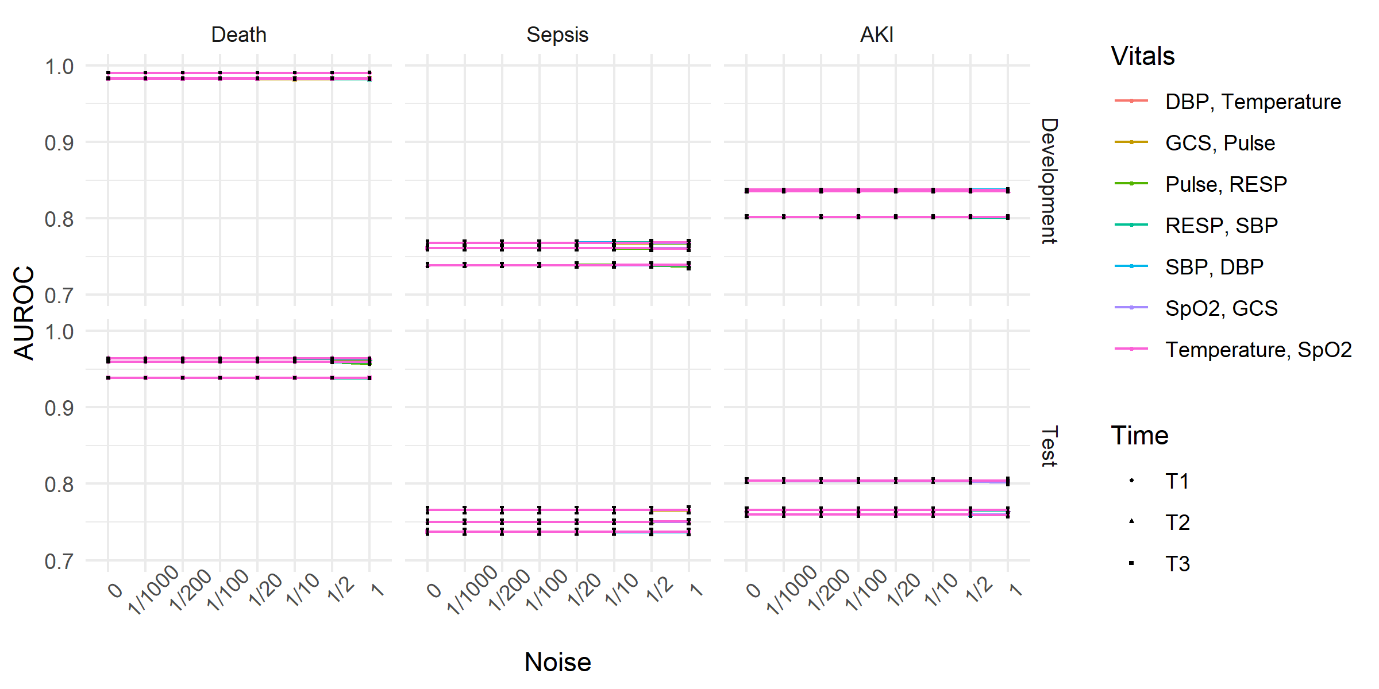

Supplement: Multimedia Appendix 7 [file medinform_v9i11e26426_app7.docx]
