# Supplementary material for: Event Prediction Model Considering Time and Input Error Using Electronic Medical Records in the Intensive Care Unit: Retrospective Study
Source: JMIR Med Inform. 2021 Nov 4;9(11):e26426. doi: 10.2196/26426 (PMC8603167; doi:10.2196/26426)

**Multimedia Appendix 8. The AUROCs of our model and non-time-series model inputted several representative values, such as last, highest, median, and lowest value of all time windows.**


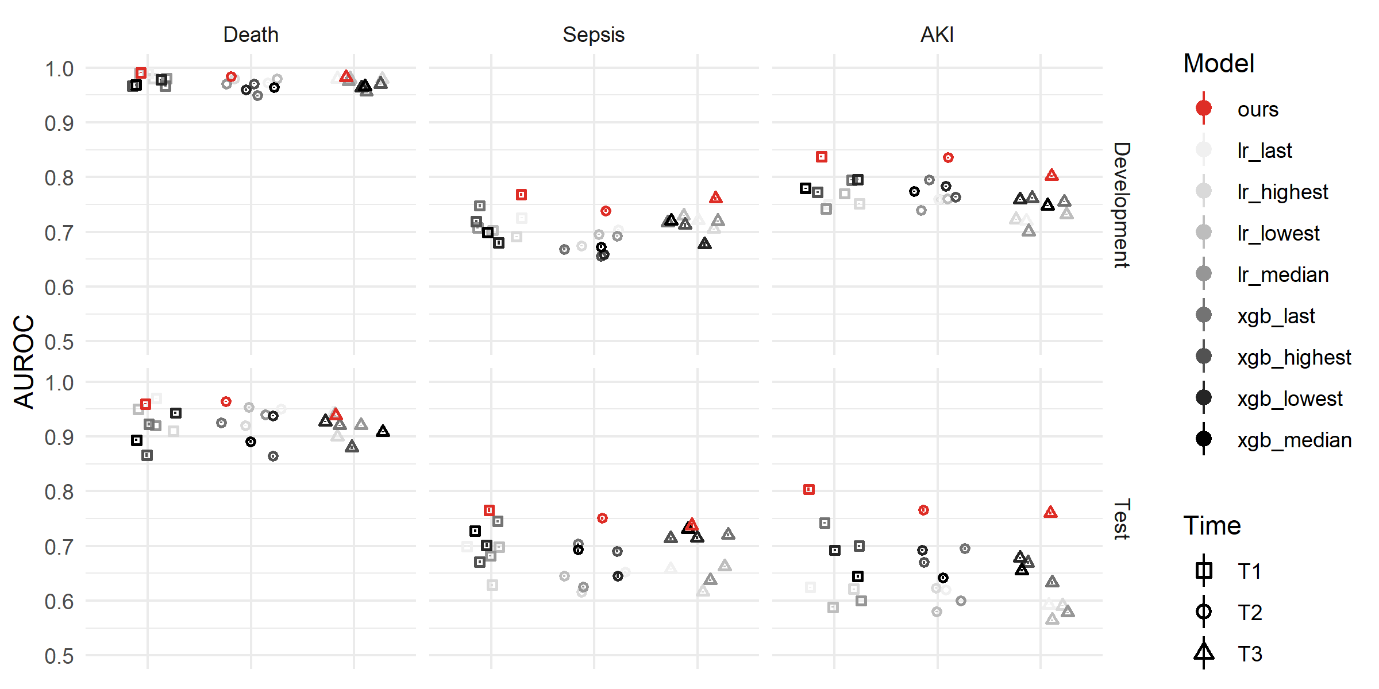

Supplement: Multimedia Appendix 8 [file medinform_v9i11e26426_app8.docx]
